# Evaluating implementation of International Health Regulations core capacities: using the Electronic States Parties Self-Assessment Annual Reporting Tool (e-SPAR) to monitor progress with Joint External Evaluation indicators

**DOI:** 10.1186/s12992-021-00720-5

**Published:** 2021-06-30

**Authors:** Ahmed Razavi, Samuel Collins, Anne Wilson, Ebere Okereke

**Affiliations:** International Health Regulations Strengthening Project, Global Public Health, Public Health England, London, UK

**Keywords:** International health regulations, Global Health security, Joint external evaluations, World Health Organization, Monitoring and evaluation, Technical support, Aid and development

## Abstract

**Background:**

The International Health Regulations (IHR) are a legally binding instrument designed to improve Global Health Security by limiting the cross boarder spread of health risks. All 196 signatories to the IHR (2005) are required to report progress towards IHR core capacity implementation through an annual multi-sectoral self-assessment process known as the State Parties Self-Assessment Annual Reporting (SPAR). This mandatory process sits alongside the voluntary, external, peer-reviewed Joint External Evaluations (JEE) as two core components of the IHR monitoring and evaluation framework. JEEs are intended to occur once every 4–5 years following a voluntary request from the member state. This means that interim monitoring of IHR core capacity compliance, can be challenging and additional data sources are required. The outputs of the SPAR process represent one such source. Although the JEE and SPAR tools are intended to be complimentary, there has been no publicly available mapping of JEE indicators to SPAR indicators in order to inform progress on IHR compliance.

**Results:**

This paper mapped JEE indicators to SPAR indicators and found a high level of correlation suggesting the SPAR process offers a method for countries and technical assistance programmes to monitor progress on IHR compliance and identify gaps in between JEE visits. However, coverage was not complete, and several gaps were identified most notably in antimicrobial resistance (AMR) and vaccinations.

**Conclusion:**

Enhancing alignment between JEE and SPAR could offer a more consistent and complete way of assessing compliance with IHR.

**Supplementary Information:**

The online version contains supplementary material available at 10.1186/s12992-021-00720-5.

## Background

The International Health Regulations (IHR 2005), a legally binding instrument, were originally adopted in 1969 to reduce the negative effects on human health and trade associated with cross-border health threats [1, 2]. Under the IHR member states were obliged to report to the World Health Organization (WHO) on outbreaks of specific infectious diseases which had the capacity to cross borders [1]. A revised edition of the IHR, characterized by an all hazards approach to human health threats was introduced in 2005 [2]. Thirteen intersectoral detection, monitoring and response core capacities were introduced to facilitate member states in the effective prevention, control and reporting of potential health threats of international significance [3].

**Fig. 1:**
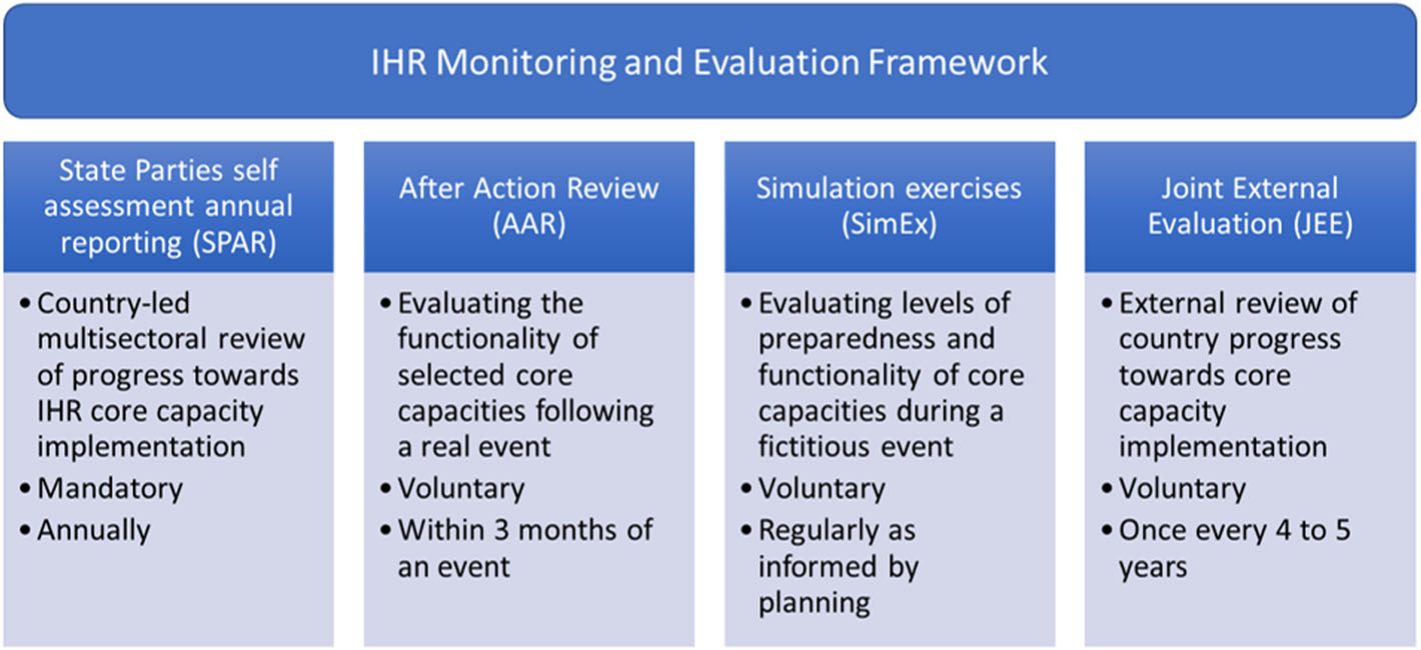
The IHR Monitoring and Evaluation Framework introduced in 2015

Through the IHR (2005) 196 member states agreed to work together to address key challenges in global health security. Under Article 54 of the IHR (2005) ‘State Parties and the Director General shall report to the Health Assembly on the implementation of these Regulations as decided by the Health Assembly. Reporting was initially achieved through State Party self-evaluation on an annual basis. Between 2010 and 2017 an IHR monitoring questionnaire was sent to IHR National Focal Points and used at least once by all member states [4]. In 2015 the 68th World Health Assembly (WHA), through report WHA68/22 Add.1, recommended expanding the way IHR compliance was assessed, initiating the development of an IHR Monitoring and Evaluation Framework (IHRMEF) [5] (Fig. 1). The IHRMEF defines a common approach to monitoring and evaluation across member states and is designed to; assist member states to self-evaluate progress toward sustainable IHR core capacity implementation; facilitate the development of plans to develop and enhance these capacities; standardise the reporting mechanism to the WHA and enable WHO to appropriately target capacity building support.

Two salient features of the IHRMEF are self-assessment annual reporting and a peer-reviewed evaluation process known as a Joint External Evaluation (JEE). These quantitative assessments sit alongside after-action reviews (AAR) and simulation exercises, qualitative processes designed to assess the real and potential functionality of member states core capacities.

**Fig. 2:**
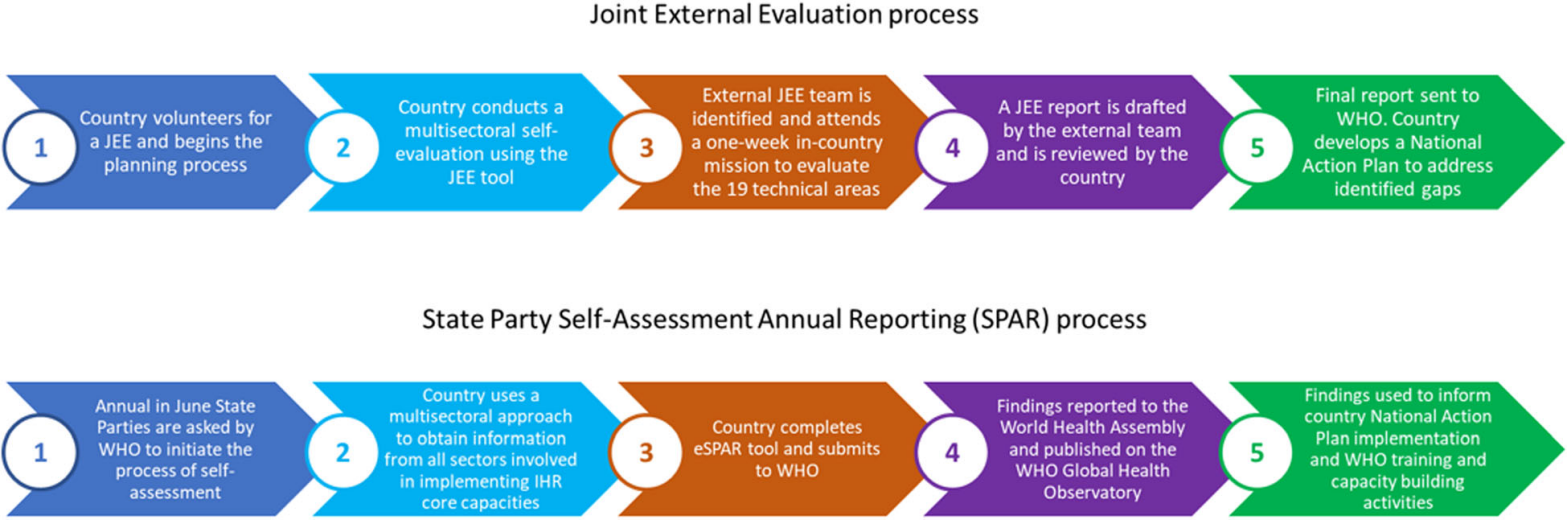
The Joint External Evaluation (JEE) and State Party Self-Assessment Annual Reporting (SPAR) process

In recognition of the need for increased accountability and transparency at an international level, the voluntary JEE process moved IHR monitoring away from simply self-reporting. The JEE is initiated at the request of a member state and begins with an initial self-assessment by the host country followed by a one-week expert mission led by WHO subject matter experts. Strengths, weaknesses, best practice and challenges in IHR core capacity implementation are identified in a collaborative process. To ensure a more transparent, independent and objective assessment of a country’s ability to deal with public health issues the JEE process is supported by the JEE tool. The JEE tool details a set of 49 indicators under 19 technical areas in order to establish a baseline assessment and was first approved in February 2016, with a second edition being released in 2018 [6]. It is intended to help countries identify gaps and target resources to those elements of the IHR that they may not be compliant with. The result of the JEE is a final report that includes scores against each of the 19 technical areas with recommendations for improvements and priorities. The JEE report informs the development of a National Action Plan for Health Security to address identified gaps. A summarised version of the JEE process is shown in Fig. 2.

Under the IHRMEF JEEs are supposed to occur every 4–5 years in member states, though in practice uptake has skewed towards low- and middle-income countries, with poor uptake amongst high-income countries [7]. In the interim, member states are requested to report annually to the WHA on their IHR capacities. Mid-year States Parties are informed to initiate the process of self-assessment and reporting to the WHA, using a multisectoral approach to obtain information from all sectors involved in implementing IHR core capacities. To facilitate this quantitative process the electronic IHR State Party Self-Assessment Annual Reporting (eSPAR) tool was updated in 2018 [4], consisting of 24 indicators for 13 IHR capacities derived from Annex 1A and 1B of IHR [2]. One to three indicators are used for each capacity and indicators are further subdivided into five elements called ‘levels of performance’. Each level of performance details a set of ‘attributes’ which must be met in order for that level to be achieved. For each indicator the members state selects the performance level that best describes its progress towards implementation. The results are submitted to WHO annually, summarised and published on the WHO Global Health Observatory alongside results from all other member states.

Evaluating progress towards IHR core capacity implementation is one indicator that can be used to monitor the impact of technical assistance programmes aimed at strengthening Global Health Security. One such initiative is the Public Health England led International Health Regulations (IHR) Strengthening Project, a UK Aid funded technical assistance programme. Taking a One Health, all hazards approach to improving national compliance with IHR, the programme aims to strengthen national, and as a result, regional and global health security by employing a triple mandate to build technical capabilities of public health institutions and public health bodies; strengthen leadership to improve multi sector coordination and; develop sustainable resilient public health systems.

Although the results of JEEs may be seen as the gold standard measurement, they occur relatively infrequently. Therefore, data from the SPAR process may be a valuable indicator for monitoring IHR compliance between JEEs. However, although the JEE and SPAR tools are intended to be complimentary, as far as the authors are aware there has been no published mapping of JEE indicators to SPAR indicators in order to inform progress on IHR compliance. As part of the PHE IHR Strengthening Projects remit to build the evidence base for effective health systems strengthening, this paper investigates how SPAR and JEE indicators correlate and explores gaps in coverage.

## Methods

Lists of JEE indicators (2nd edition, [6]) and SPAR indicators [4] were obtained from the WHO website. Using SPAR indicators as the baseline, JEE indicators were mapped to these based on the descriptions to each indicator provided by the WHO. Once each SPAR indicator had been mapped to a JEE indicator (where possible), remaining JEE indicators were identified and any further correlations were explored. The resulting mapping was re-checked at this stage and each mapping was assigned categories based on the following definitions:

- Direct match – SPAR indicator(s) are captured in their entirety by JEE indicator(s)
- Close match – SPAR indicator(s) are captured in their entirety by the JEE indicator(s) but there may be additional details in the JEE indicator that are not captured by the SPAR indicator.
- No match – No clear correlation between SPAR indicator and JEE indicator.

A full list of both JEE and SPAR indicators can be found in supplementary Tables 1 and 2.

## Results

The JEE indicators were mapped to SPAR indicators as shown in Table 1. 23 SPAR indicators were mapped in some form to 41 JEE indicators.

**Table 1:**
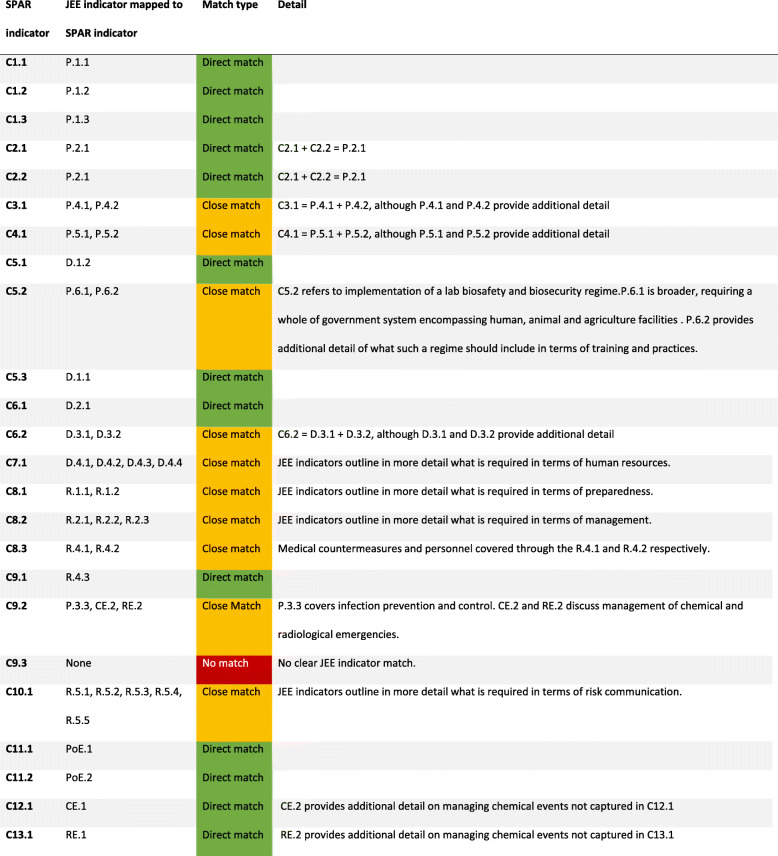
The complete mapping of JEE indicator to SPAR indicator

Of the mapped SPAR indicators, 13 were considered to be ‘direct matches’, with 10 ‘close matches’ (Table 1). Only one SPAR indicator (C9.3 - access to essential healthcare) had no mapped JEE indicator. Ten JEE indicators had no clear match with the SPAR indicators identified, though in some cases these indicators may be providing additional detail not captured in SPAR.

Direct matches included indicators on legislation, IHR coordination, points of entry and emergencies. Close matches included indicators on specific issues such as zoonotic disease and food safety, as well as emergency frameworks and risk communication. These were not classified as direct matches as the JEE indicators outlined more detailed requirements for meeting these indicators.

The outstanding JEE indicators that were not mapped to any SPAR indicators concerned anti-microbial resistance (AMR), immunisation linking public health and security authorities and additional detail on laboratory systems and surveillance systems.

## Discussion

In this paper we examined the correlation between two of the fundamental tools underlying the quantitative aspects of the IHR Monitoring and Evaluation Framework (IHRMEF), the State Self-Assessment and Reporting (SPAR) tool and the Joint External Evaluation (JEE) tool. JEE tool indicators were mapped to SPAR indicators and the degree of match assessed. The majority (23 of 24) SPAR indicators mapped either closely or directly with JEE indicators demonstrating a high level of correlation between the two. Using SPAR indicators to monitor progress on JEE indicators could therefore be a feasible option for many of the JEE domains.

There are however gaps around AMR (JEE indicators P.3.1, P.3.2 and P3.4) and vaccinations (JEE indicators P.7.1 and P.7.2). These are important gaps particularly given the global significance of AMR and the possibility that the emergence and spread of antimicrobial-resistant bacteria (particularly pan-resistant strains) could represent a public health emergency of international concern (PHEIC) [7].

The SPAR tool also provides limited usefulness in indicators relating to laboratory systems. Two out of four JEE indicators were mapped with D1.3 ‘Effective national diagnostic network’ and D1.4 ‘Laboratory quality system’ unable to be mapped to the JEE indicators. Likewise, for surveillance, the SPAR indicator does not cover JEE D.2.2 (use of electronic tools) or D.2.3 (analysis of surveillance data). This may have been a pragmatic decision taken to reduce the burden of reporting against SPAR indicators and simplify the process. The SPAR indicator that could be considered missing within the JEE indicators is SPAR C9.3 ‘access to essential health services’. This could be considered part of R.4.3 ‘Case management procedures implemented’, though there is no explicit mention of ensuring access to essential medical services within the JEE tool. Access to essential health services can be a key determinant of health systems performance and resilience and has been shown to be a key component of health security. For example, it is reported that the EVD outbreak in Sierra Leone resulted in higher mortality from endemic diseases and maternal and child health conditions as a consequence of the collapse of the health system under the pressures of the outbreak. Therefore, measuring access to essential health services warrants explicit inclusion within any future edition of JEE.

It should be noted that neither JEE nor SPAR measure all aspects of Global Health Security. A potential gap in the IHRMEF exists around knowledge sharing during a public health emergency [8] the importance of which has been driven home during the COVID-19 pandemic, particularly information generated from non-health services, example international trade and procurement, transport. This is important both internationally and within countries, ensuring that data on contributory non-health factors is shared with agencies responsible for coordinating emergency response, including the national public health institute (or equivalent). Additionally, the JEE and SPAR both measure IHR compliance and preparedness at a defined point of time. It is well known that in response to PH events, capacity can be rapidly expanded / enhanced in response to the emergency and thus may change significantly from what it was at the time of the last measurement. With dynamic changes being made to health systems during the COVID-19 pandemic potentially altering compliance with IHR, JEE and SPAR scoring must be seen as a snapshot of the health system at the time of the assessment.

Using the results of SPAR offers a method for countries to monitor progress on IHR compliance and identify gaps in between JEE visits. Such as approach could also be viable for technical aid donors, such as Public Health England’s IHR Strengthening Project, to evaluate whether improvements are being made during the life cycle of the project, though it must be noted that correlation does not imply causation. Using the mapping above, correlations can be drawn from the start of the project and any subsequent improvement in indicator status over time. However, this approach is not without limitations. The SPAR tool contains self-reported data and as a result this may lead to social desirability bias in the results. Though the JEE indicators are also initially self-reported, these are then triangulated by an external team and as a result can be considered a more reliable representation of IHR compliance. Additionally, in the 10 ‘close matches’ the SPAR indicators tended to require less detail than the respective JEE indicators, potentially resulting in a lower threshold of IHR compliance to meet the SPAR indicator when compared to the more detailed JEE indicator. An example of this is SPAR indicator C.10 ‘Capacity for emergency risk communications’ which maps to five separate JEE indicators covering specific types of communications such as ‘addressing perceptions, risky behaviours and misinformation’ (JEE indicator R.5.5) and ‘public communication for emergencies’ (JEE indicator R.5.3).

Given the current landscape of global health security in the midst of the COVID-19 pandemic and the ongoing review of IHR in response to COVID-19 it is worth reflecting on JEE and SPAR in light of our mapping to inform future decision-making. First, the limitations highlighted above could have important policy implications. Identified gaps in access to essential health care (many countries are struggling with this in the COVID-19 pandemic), AMR, vaccination and laboratory and surveillance systems raise significant issues regarding pandemic preparedness. In its interim report [9] the Panel on Pandemic Preparedness and Response concluded that the current global pandemic alert system is ‘slow, cumbersome and indecisive’ and requires significant strengthening to enable flexibility and ‘reaction at speed’. WHOs response relies on robust national surveillance and response capacity, in compliance with IHR, therefore the gaps identified here should be examined as a priority, particularly in light of the global response to COVID-19. Second, SPAR was updated in 2018 in order to provide a more comprehensive self-assessment of IHR compliance in between JEEs. The indicators created cover most JEE domains to a lesser degree of detail, which is to be expected for an interim assessment of progress. However, there is still scope to make the SPAR indicators more robust and evidence-based, depending on the appetite for this given a limited pool of resources for some countries.

Third, SPAR indicators could also be more closely aligned with JEE indicators regardless of the level of detail. Having the same overarching indicators for both SPAR and JEE with sub-indicators for JEE providing an additional level of detail could be a way of entirely aligning the WHO’s IHR Monitoring and Evaluation process. This would also remove the need for undertaking any mapping process in order to determine how changes in SPAR scoring would impact JEE performance. Additionally, given the many competing demands for health systems having a shorter and well aligned tool covering both SPAR and JEE should have greater utility.

Finally, it should be noted that although Kandel et al [8] showed good correlation between SPAR and JEE scores for IHR capacity, the true power of the JEE and SPAR process to predict country-level preparedness for public health emergencies and the degree to which good correlation between SPAR and JEE correlates with effective preparedness and response remains undefined. Neither JEE nor SPAR may fully assess preparedness as other factors outside of IHR compliance such as political engagement and health system resilience will impact preparedness and response. Findings from the current COVID-19 pandemic may prove enlightening in this regard. Global understanding of factors that contribute to emergency preparedness is growing, therefore the tools for measuring preparedness will continue to evolve and will be influenced by policy positions of the global health security leadership. The differences highlighted in this paper hint at the challenges of norm building through international institutions and having agreement across all WHO member states on a shared approach.

## Conclusion

The high level of correlation between SPAR and JEE indicators provides reassurance that the two reporting tools are complimentary and somewhat interchangeable. Although the true power of the SPAR and JEE processes to predict country-level preparedness for public health emergencies is still unknown SPAR is a useful process and can be used to demonstrate progress with IHR compliance in between JEE visits. Countries and projects aiming to increase IHR compliance could use SPAR to demonstrate the broad impact of their approaches. This should be complimented by a more detailed evaluation of progress on each indicator. Future work could include mapping the PHE IHR Strengthening Project logical framework (logframe) to the SPAR tool in order to assess compatibility between the two and potentially monitor the impact meeting logframe indicators has on IHR compliance.

## Supplementary Information

**Additional file 1.**

## Data Availability

All data is available from the WHO website and all results are presented within this manuscript.
