# Supplementary material for: Evaluating implementation of International Health Regulations core capacities: using the Electronic States Parties Self-Assessment Annual Reporting Tool (e-SPAR) to monitor progress with Joint External Evaluation indicators
Source: Global Health. 2021 Jun 30;17:69. doi: 10.1186/s12992-021-00720-5 (PMC8243291; doi:10.1186/s12992-021-00720-5)
Supplement: Supplementary file 1 — Additional file 1. [file 12992_2021_720_MOESM1_ESM.docx]

### Supplementary material

Table S1: SPAR indicators

| **SPAR Topic** | **SPAR Indicator** | **SPAR Description** |
| --- | --- | --- |
| C1. LEGISLATION AND FINANCING | C1.1 | Legislation, laws, regulations, policy, administrative requirements or other government instruments to implement the IHR |
|  | C1.2 | Financing for the implementation of IHR capacities |
|  | C1.3 | Financing mechanism and funds for timely response to public health emergencies |
| C2. IHR COORDINATION AND NATIONAL IHR FOCAL POINT FUNCTIONS | C2.1 | National IHR Focal Point functions under IHR |
|  | C2.2 | Multisectoral IHR coordination mechanisms |
| C3. ZOONOTIC EVENTS AND THE HUMAN–ANIMAL INTERFACE | C3.1 | Collaborative effort on activities to address zoonoses |
| C4. FOOD SAFETY | C4.1 | Multisectoral collaboration mechanism for food safety events |
| C5. LABORATORY | C5.1 | Specimen referral and transport system |
|  | C5.2 | Implementation of a laboratory biosafety and biosecurity regime |
|  | C5.3 | Access to laboratory testing capacity for priority diseases |
| C6. SURVEILLANCE | C6.1 | Early warning function: indicator-and event-based surveillance |
|  | C6.2 | Mechanism for event management (verification, risk assessment, analysis, investigation) |
| C7. HUMAN RESOURCES | C7.1 | Human resources for the implementation of IHR capacities |
| C8. NATIONAL HEALTH EMERGENCY FRAMEWORK | C8.1 | Planning for emergency preparedness and response mechanism |
|  | C8.2 | Management of health emergency response operations |
|  | C8.3 | Emergency resource mobilization |
| C9. HEALTH SERVICE PROVISION | C9.1 | Case management capacity for IHR relevant hazards |
|  | C9.2 | Capacity for infection prevention and control and chemical and radiation decontamination |
|  | C9.3 | Access to essential health services |
| C10. RISK COMMUNICATION | C10.1 | Capacity for emergency risk communications |
| C11. POINTS OF ENTRY | C11.1 | Core capacity requirements at all times for designated airports, ports and ground crossings |
|  | C11.2 | Effective public health response at points of entry |
| C12. CHEMICAL EVENTS | C12.1 | Resources for detection and alert |
| C13. RADIATION EMERGENCIES | C13.1 | Capacity and resources |

Table S2: JEE indicators

| **JEE Indicator Topic** | **JEE Indicator** | **JEE Description** |
| --- | --- | --- |
| National legislation, policy and financing | P.1.1 | The State has assessed, adjusted and aligned its domestic legislation, policies and administrative arrangements in all relevant sectors, to enable compliance with the IHR |
|  | P.1.2 | Financing is available for the implementation of IHR capacities |
|  | P.1.3 | A financing mechanism and funds are available for timely response to public health emergencies |
| IHR coordination, communication and advocacy | P.2.1 | A functional mechanism is established for the coordination and integration of relevant sectors in the implementation of IHR |
| Antimicrobial resistance | P.3.1 | Effective multisectoral coordination on AMR |
|  | P.3.2 | Surveillance of AMR |
|  | P.3.3 | Infection prevention and control |
|  | P.3.4 | Optimize use of antimicrobial medicines in human and animal health and agriculture |
| Zoonotic diseases | P.4.1 | Coordinated surveillance systems in place in the animal health and public health sectors for zoonotic diseases/pathogens identified as joint priorities |
|  | P.4.2 | Mechanisms for responding to infectious and potential zoonotic diseases established and functional |
| Food safety | P.5.1 | Surveillance systems in place for the detection and monitoring of foodborne diseases and food contamination |
|  | P.5.2 | Mechanisms are established and functioning for the response and management of food safety emergencies |
| Biosafety and biosecurity | P.6.1 | Whole-of-government biosafety and biosecurity system in place for all sectors (including human, animal and agriculture facilities) |
|  | P.6.2 | Biosafety and biosecurity training and practices in all relevant sectors (including human, animal and agriculture) |
| Immunisation | P.7.1 | Vaccine coverage (measles) as part of national programme |
|  | P.7.2 | National vaccine access and delivery |
| National laboratory system | D.1.1 | Laboratory testing for detection of priority diseases |
|  | D.1.2 | Specimen referral and transport system |
|  | D.1.3 | Effective national diagnostic network |
|  | D.1.4 | Laboratory quality system |
| Surveillance | D.2.1 | Surveillance systems |
|  | D.2.2 | Use of electronic tools |
|  | D.2.3 | Analysis of surveillance data |
| Reporting | D.3.1 | System for efficient reporting to FAO, OIE and WHO |
|  | D.3.2 | Reporting network and protocols in country |
| Human resources (animal and human health sectors) | D.4.1 | An up-to-date multisectoral workforce strategy is in place |
|  | D.4.2 | Human resources are available to effectively implement IHR |
|  | D.4.3 | In-service trainings are available |
|  | D.4.4 | FETP or other applied epidemiology training programme is in place |
| Preparedness | R.1.1 | Strategic emergency risk assessments conducted, and emergency resources identified and mapped |
|  | R.1.2 | National multisectoral multihazard emergency preparedness measures, including emergency response plans, are developed, implemented and tested |
| Emergency response operations | R.2.1 | Emergency response coordination |
|  | R.2.2 | Emergency operations centre (EOC) capacities, procedures and plans |
|  | R.2.3 | Emergency exercise management programme |
| Linking public health and security authorities | R.3.1 | Public health and security authorities (e.g. law enforcement, border control, customs) linked during a suspect or confirmed biological, chemical or radiological event |
| Medical countermeasures and personnel deployment | R.4.1 | System in place for activating and coordinating medical countermeasures during a public health emergency |
|  | R.4.2 | System in place for activating and coordinating health personnel during a public health emergency |
|  | R.4.3 | Case management procedures implemented for IHR relevant hazards |
| Risk communication | R.5.1 | Risk communication systems for unusual/ unexpected events and emergencies |
|  | R.5.2 | Internal and partner coordination for emergency risk communication |
|  | R.5.3 | Public communication for emergencies |
|  | R.5.4 | Communication engagement with affected communities |
|  | R.5.5 | Addressing perceptions, risky behaviours and misinformation |
| Points of entry | PoE.1 | Routine capacities established at points of entry |
|  | PoE.2 | Effective public health response at points of entry |
| Chemical events | CE.1 | Mechanisms established and functioning for detecting and responding to chemical events or emergencies |
|  | CE.2 | Enabling environment in place for management of chemical events |
| Radiation Emergencies | RE.1 | Mechanisms established and functioning for detecting and responding to radiological and nuclear emergencies |
|  | RE.2 | Enabling environment in place for management of radiological and nuclear emergencies |
